# Association of smoking cessation with late-life frailty risk: the Singapore Chinese health study

**DOI:** 10.3389/fragi.2026.1870256

**Published:** 2026-09-17

**Authors:** Ainsley Ryan Yan Bin Lee, Huiqi Li, Kevin Yiqiang Chua, Woon-Puay Koh

**Affiliations:** 1 Department of Medicine, Yong Loo Lin School of Medicine, National University of Singapore, Singapore; 2 Healthy Longevity Translational Research Programme, Yong Loo Lin School of Medicine, National University of Singapore, Singapore; 3 Institute for Human Development and Potential, Agency for Science, Technology and Research (A*STAR), Singapore, Singapore

**Keywords:** frailty, healthy ageing, older adults, smoking cessation, tobacco use

## Abstract

**Objective:**

To examine whether smoking cessation, in terms of duration and age at quitting, was associated with late-life physical frailty.

**Design:**

Prospective population-based cohort study.

**Setting:**

Chinese living in Singapore.

**Participants:**

We analyzed data from 4,574 men (mean baseline age 52.7 years, range 45–65 years) enrolled in the Singapore Chinese Health Study.

**Measurements:**

Participants self-reported smoking behaviors at four time-points over a 20-year follow-up. Physical frailty was assessed in late life (mean age 73.6 years, range 65–88 years) using a modified Cardiovascular Health Study phenotype, incorporating weakness, slowness, exhaustion and weight loss. Multivariable logistic regression was used to estimate odds ratios (OR) and 95% confidence intervals (CI) for association with frailty.

**Results:**

Persistent smokers had higher odds of frailty compared to never smokers (OR: 1.34; 95% CI: 1.01–1.76). Those who had quit smoking within 10 years before frailty assessment also had increased odds (OR = 1.33; 95% CI: 0.86–2.01), albeit not statistically significant. The OR (95% CI) was attenuated to 1.08 (0.76–1.51) for those quitting 10–20 years and 0.97 (0.59–1.55) for those quitting >40 years. Those who quit smoking by age 60 years had similar odds relative to never smokers: OR was 1.05 (0.74–1.48) for quitting at ages 50–60 years and further reduced to 0.86 (0.57–1.25) for quitting at ages <40 years.

**Conclusion:**

While persistent smoking was associated with increased odds of late-life physical frailty, smoking cessation for at least 10 years, or before the age of 60 years, could mitigate this risk substantially.

## 1 Introduction

Frailty is a multifaceted syndrome characterized by a decline in physical strength and function, physiologic equilibrium, and the ability to maintain homeostasis. This age-related risk is influenced by a combination of genetic, biological, and environmental factors (Fried et al., 2004; Clegg et al., 2013). Since the landmark paper by Fried et al., who defined the “frailty phenotype” based on specific clinical criteria (unintentional weight loss, weakness, self-reported exhaustion, slow walking speed, and low physical activity) (Morley et al., 2013; Kojima, 2015; Fried et al., 2001), frailty has been found to be a significant predictor of disability, hospitalization, and mortality (Morley et al., 2013; Kojima, 2015; Fried et al., 2001).

Smoking is a well-established risk factor for numerous chronic diseases, including cardiovascular disease (Lakier, 1992; Burns, 2003), chronic obstructive pulmonary disease (Forey et al., 2011), and numerous cancers (Caliri et al., 2021; Warren and Cummings, 2013; Gandini et al., 2008). Recently, it has also been recognized to play a role in the initiation and progression of frailty. The physiological mechanisms linking smoking to frailty are multifaceted, encompassing systemic inflammation (Goncalves et al., 2011; Gale et al., 2013), oxidative stress (Caliri et al., 2021), and declines in skeletal muscle mass and function (Degens et al., 2015).

Several observational studies have reported the association between smoking and physical frailty. A systematic review and meta-analysis of prospective studies found that current smokers had a 63% higher risk of incident frailty than lifetime abstainers, whereas former smokers did not show a statistically significant excess risk (Amiri and Behnezhad, 2019). What remains unclear, however, is whether frailty risk among former smokers varies by how long ago they quit or when they quit (e.g., earlier vs. later in life), because prospective evidence addressing cessation duration and age at cessation is limited. One analysis from the English Longitudinal Study of Ageing (ELSA) followed frailty trajectories over 16 years in 2,600 former smokers (grouped by time since cessation) and 2,061 never smokers (Kojima et al., 2025). Frailty levels were generally lower among those who had quit for longer, and those with ≥41 years since cessation showed the lowest frailty trajectory; yet former smokers still had higher frailty than never smokers. Notably, the rate of change in frailty over time did not differ by cessation duration, leaving uncertainty about whether quitting earlier altered frailty progression rather than simply being associated with lower baseline frailty (Kojima et al., 2025). In contrast, data from the Survey on Health, Ageing, and Retirement in Europe (SHARE) suggested that the odds of worsening frailty were 16% higher in current smokers versus never smokers, but only a nonsignificant 7% higher in former smokers (Etman et al., 2015). However, SHARE did not assess whether the timing or duration of cessation modified frailty risk, so the extent to which quitting earlier or remaining abstinent longer could meaningfully reduce frailty risk remains unresolved.

We therefore investigated, in a prospective population-based cohort of Chinese men in Singapore, whether the odds of late-life physical frailty differed according to duration since smoking cessation and age at smoking cessation. Repeated assessment of smoking status over four study visits allowed us to systematically classify never smokers, persistent smokers, and former smokers prospectively, thereby effectively addressing this research gap.

## 2 Methods

### 2.1 Study population

This study was nested within the Singapore Chinese Health Study, a population-based prospective cohort study that recruited 63,257 participants (27,959 men and 35,298 women) aged 45–74 years old between April 1993 and December 1998. All participants were of Chinese ethnicity, belonging to either the Hokkien or Cantonese dialect groups, and resided in government-built housing estates, which accommodated 86% of Singapore’s population at the time of recruitment. Further details regarding this cohort study have been published previously (Hankin et al., 2001).

After recruitment, all consenting participants were re-contacted for follow-up interviews every five to 6 years, with the first follow-up interviews being conducted from 1999 to 2004, the second follow-up interviews from 2006 to 2010, and the third follow-up interviews from 2014 to 2017. Ethical approval to conduct this study was granted by the Institutional Review Board at the National University of Singapore. Informed written consent was obtained from all study participants.

### 2.2 Assessment of smoking habits and other covariates

At baseline, in-person interviews were conducted by trained interviewers using a structured questionnaire to collect information about sociodemographic factors (age, sex, dialect group, and education level), height, weight, lifestyle factors (physical activity, alcohol drinking, usual diet, and sleep duration), and physician-diagnosed medical history of chronic diseases (hypertension, ischemic heart disease, stroke and diabetes). Dietary intake over the past year was measured using a validated food-frequency questionnaire, and diet quality was assessed based on adherence to the Dietary Approaches to Stop Hypertension (DASH) diet, focusing on high intake of fruits, vegetables, nuts and legumes, low-fat dairy, and whole grains, while limiting red/processed meats, and sugary beverages (Talaei et al., 2019; Lai et al., 2024). Body mass index (BMI) was derived using each participant’s self-reported height and weight.

Data related to smoking habits were collected by asking participants whether they ever smoked “at least one cigarette a day for 1 year or longer”. Participants who answered “no” were categorized as never smokers, those who answered “yes, but I quit smoking” were categorized as former smokers, and those who answered “yes, and I currently smoke” were categorized as current smokers. At follow-up interviews, participants were asked similar questions to update their smoking status. In all surveys, we also obtained information on the number of cigarettes smoked per day and age of smoking initiation for smokers, and if they quit, the number of years since quitting.

Participants were classified as never smokers if they reported never smoking at baseline and at all three follow-up interviews. Persistent smokers were participants who reported current smoking at baseline and at all three follow-up interviews. Former smokers were participants who indicated a history of smoking either at baseline or during a follow-up interview, and did not report resuming smoking at any later time point. Participants who relapsed after an initial quitting attempt and resumed smoking were excluded from the analysis. For former smokers, age at quitting was determined by subtracting years since quitting from their reported age at the interview; duration since cessation was calculated as the difference between age at frailty assessment and age at quitting smoking.

### 2.3 Assessment of physical frailty in late life

Physical frailty was assessed during follow-up 3 interviews (2014–2017) using a modified Cardiovascular Health Study frailty phenotype (Morley et al., 2013; Fried et al., 2001). Four criteria were included: weight loss, exhaustion, slowness, and weakness; low physical activity was excluded as we did not collect relevant data on level of physical activity. We had previously published risk factors of physical frailty using this definition from these four criteria in this cohort (Ch et al., 2026; Chua et al., 2024): 1) weight loss was defined as having lost ≥10% of body weight between follow-up 2 (2006–2010) and follow-up 3 (2014–2017); 2) exhaustion was defined by “No” to the question, “Are you full of energy?”; 3) slowness was defined by being in the slowest sex-specific quintile in the Timed Up-and-Go test; 4) weakness was determined by being in the weakest sex-specific quintile of handgrip strength. Participants meeting at least two criteria were classified as physically frail. Compared with the original five-component phenotype, this definition could classify some participants who would otherwise be considered pre-frail as frail. We therefore repeated a sensitivity analysis using a stricter definition of at least 3 of the 4 available criteria.

### 2.4 Statistical analyses

We did not include women in this study due to the low prevalence of smoking among the women in this cohort (only 3.0% were smokers at baseline). A total of 5,186 men in this cohort had complete data on smoking habits at all surveys and completed assessment for physical frailty at follow-up 3 interviews. Of the initial sample, 175 men were excluded for exceeding the age of 65 years at baseline, 142 men were excluded for being younger than 65 years at the time of frailty assessment, and 295 men were excluded due to resuming smoking after initially quitting. This resulted in a total of 4,574 men included in the present analysis. The final sample represented 16.4% of the 27,959 men recruited at baseline.

Differences in characteristics across groups of smoking cessation were tested using one-way ANOVA for continuous variables or chi-square test for categorical variables. Multivariate logistic regression models were used to calculate odds ratios (ORs) and 95% confidence intervals (95% CIs) of the associations between elements of smoking cessation and physical frailty at follow-up 3. Model 1 was adjusted for age at assessment of physical frailty, dialect group (Hokkien, Cantonese), and levels of education (no formal, primary, secondary school and higher). Model 2 was additionally adjusted for factors that were either established risk factors of physical frailty in the literature and/or in our cohort (Lai et al., 2024; Chua et al., 2023; Dai et al., 2025; Abd Ghafar et al., 2022; Song et al., 2024). These factors collected at baseline included BMI (<18.5, 18.5–22.9, 23–27.4, ≥27.5 kg/m^2^), alcohol consumption (non-drinker, monthly drinker, weekly drinker, daily drinker), sleep duration (≤5, 6–8, ≥9 h/day), DASH scores (quartiles), physical activity (no physical activity, 0.5–3.9 h a week, ≥4 h/week), caffeine intake (quartiles), and medical history of hypertension, diabetes, cardiovascular diseases at baseline. Additionally, we also adjusted for data on BMI, alcohol, sleep duration, DASH scores, and the same medical history collected at follow-up 3. To examine potential health-related smoking cessation, we reclassified former smokers who had quit within 2 years before frailty assessment as persistent smokers and repeated the analyses after excluding participants with hypertension, diabetes, ischemic heart disease, or stroke at baseline. A P-value for trend was calculated among former smokers by assigning ordinal scores to categories of increasing duration since cessation (<10, 10–20, 20–30, 30–40, >40 years). Finally, we repeated the analyses using frailty defined as at least 3 of the 4 available criteria.

All analyses were conducted in R 4.5.0. All *P*-values were two-sided, and a *P*-value of <0.05 was considered statistically significant.

## 3 Results

Among the 4,574 men included in the analysis, 2,527 (55.3%) were never smokers, 1,400 (30.6%) were former smokers, and 647 (14.1%) were persistent smokers. Mean age was 52.7 years at baseline, 58.3 years at follow-up 1, 65.1 years at follow-up 2, and 73.6 years at follow-up 3, when frailty was assessed. Baseline characteristics (Table 1) differed across smoking groups for all variables except sleep duration. Compared to never smokers, persistent smokers had lower education levels, lower baseline DASH scores, higher caffeine intake, more daily alcohol use, and less physical activity. Former smokers were the oldest at both baseline and frailty assessment, and had the highest prevalence of baseline hypertension, diabetes, and cardiovascular disease compared to never smokers and persistent smokers.

**TABLE 1:** Baseline characteristics of participants according to smoking status (n = 4,574).

Characteristic	Total	Never smokers	Former smokers	Persistent smokers	*P* value
Number of participants	4574	2527	1400	647	-
Age at baseline, y	52.7 ± 5.1	52.4 ± 5.1	53.6 ± 5.2	52.3 ± 5.0	<0.001
Age at frailty assessment, y	73.6 ± 5.3	73.2 ± 5.3	74.4 ± 5.4	73.2 ± 5.0	<0.001
BMI, kg/m^2^	23.2 ± 3.2	23.3 ± 3.2	23.4 ± 3.2	22.4 ± 3.2	<0.001
Dialect group	​	​	​	​	<0.001
Hokkien	2325 (50.8)	1210 (47.9)	726 (51.9)	389 (60.1)	​
Cantonese	2249 (49.2)	1317 (52.1)	674 (48.1)	258 (39.9)	​
Level of education	​	​	​	​	<0.001
No formal education	169 (3.7)	62 (2.5)	62 (4.4)	45 (7.0)	​
Primary	1979 (43.3)	886 (35.1)	742 (53.0)	351 (54.3)	​
Secondary or higher	2426 (53.0)	1579 (62.5)	596 (42.6)	251 (38.8)	​
DASH score	20.5 ± 4.7	21.3 ± 4.7	20.0 ± 4.6	18.7 ± 4.3	<0.001
Caffeine intake	158.0 ± 112.1	138.7 ± 103.5	169.2 ± 108.7	209.4 ± 130.6	<0.001
Alcohol consumption	​	​	​	​	<0.001
Non-drinker	3009 (65.8)	1759 (69.6)	899 (64.2)	351 (54.3)	​
Monthly drinker	601 (13.1)	334 (13.2)	187 (13.4)	80 (12.4)	​
Weekly drinker	736 (16.1)	368 (14.6)	231 (16.5)	137 (21.2)	​
Daily drinker	228 (5.0)	66 (2.6)	83 (5.9)	79 (12.2)	​
Sleep duration	​	​	​	​	0.391
≤5 h	261 (5.7)	151 (6.0)	74 (5.3)	36 (5.6)	​
6–8 h	4111 (89.9)	2277 (90.1)	1254 (89.6)	580 (89.6)	​
≥9 h	202 (4.4)	99 (3.9)	72 (5.1)	31 (4.8)	​
Physical activity	​	​	​	​	<0.001
No physical activity	2243 (49.0)	1142 (45.2)	731 (52.2)	370 (57.2)	​
0.5–3.9 h a week	1437 (31.4)	872 (34.5)	405 (28.9)	160 (24.7)	​
≥4 h a week	894 (19.5)	513 (20.3)	264 (18.9)	117 (18.1)	​
Hypertension	​	​	​	​	<0.001
No	3695 (80.8)	2012 (79.6)	1090 (77.9)	593 (91.7)	​
Yes	879 (19.2)	515 (20.4)	310 (22.1)	54 (8.3)	​
Diabetes	​	​	​	​	<0.001
No	4351 (95.1)	2417 (95.6)	1304 (93.1)	630 (97.4)	​
Yes	223 (4.9)	110 (4.4)	96 (6.9)	17 (2.6)	​
Cardiovascular diseases	​	​	​	​	<0.001
No	4449 (97.3)	2470 (97.7)	1343 (95.9)	636 (98.3)	​
Yes	125 (2.7)	57 (2.3)	57 (4.1)	11 (1.7)	​

Abbreviations: BMI, body mass index; DASH, dietary approaches to stop hypertension.

Data are mean ± SD, or n (%). Between-group differences were assessed using one-way ANOVA, for continuous variables and the chi-square test for categorical variables.

After an average follow-up of 20.9 years, a total of 629 (13.8%) men met the criteria of physical frailty. Participants were first analyzed by the duration of time elapsed since smoking cessation (Table 2). In fully adjusted Model 2, persistent smokers had an elevated OR (95% CI) of 1.34 (1.01–1.76) for frailty compared to never smokers. Among former smokers, quitting for less than 10 years before frailty assessment was linked to a higher OR (95% CI) of 1.33 (0.86–2.01), though this was not statistically significant. Among participants who had quit smoking for more than 10 years, the ORs (ranging from 0.85 to 1.08) were attenuated and close to null across all time-since-quitting categories, with risk estimates that were no longer statistically distinguishable from those of never smokers. In addition, there was no statistically significant dose-dependent or stepwise trend of lower risk with longer duration since cessation (P for trend = 0.112) (Supplementary Table S1).

**TABLE 2:** Association of duration since smoking cessation with late-life physical frailty (n = 4,574).

Smoking status	Cases/total	Mean age at baseline (years)	Mean age at frailty assessment (years)	Mean age at quitting smoking (years)	Mean duration quit (years)	Model 1	Model 2
						Or (95% CI)	Or (95% CI)
Never smoker	300/2527	52.4	73.2	Not applicable	Not applicable	1.00	1.00
Quitting >40 years	26/146	54.4	76.2	34.3	42.0	1.02 (0.63–1.60)	0.97 (0.59–1.55)
Quitting 30–40 years	51/385	54.1	74.2	38.6	35.6	0.93 (0.66–1.29)	0.85 (0.60–1.19)
Quitting 20–30 years	49/328	53.5	74.4	49.4	25.1	1.01 (0.71–1.42)	0.91 (0.63–1.29)
Quitting 10–20 years	55/340	53.4	74.5	59.8	14.7	1.12 (0.80–1.55)	1.08 (0.76–1.51)
Quitting <10 years	36/201	52.4	73.4	67.5	5.8	1.47 (0.97–2.16)	1.33 (0.86–2.01)
Persistent smoker	112/647	52.3	73.2	Not applicable	Not applicable	1.43 (1.11–1.84)	1.34 (1.01–1.76)

Model 1 was adjusted for age at frailty assessment, dialect group, and level of education.

Model 2 was further adjusted for body mass index (baseline, follow-up 3, categorical), alcohol drinking (baseline, follow-up 3, categorical), physical activity (baseline), sleep duration (baseline, follow-up 3, categorical), DASH (baseline, follow-up 3 in ordinal quartiles), caffeine (baseline, in ordinal quartiles), and medical history of hypertension, diabetes, cardiovascular diseases (baseline, follow-up 3).

Participants were then analyzed by their age at smoking cessation (Table 3). In these analyses, those who quit smoking at age 60 years or older had a non-significantly increased OR (95% CI) of 1.20 (0.87–1.63) for late-life physical frailty compared to never smokers. Conversely, those who quit smoking before age 60 years had risk estimates near or below null (ORs ranging from 0.84 to 1.05), with all corresponding 95% CIs encompassing 1.00.

**TABLE 3:** Association of age at smoking cessation with late-life physical frailty (n = 4,574).

Age at quitting	Cases/total	Mean age at baseline (years)	Mean age at frailty assessment (years)	Mean age at quitting smoking (years)	Mean duration quit (years)	Model 1	Model 2
						Or (95% CI)	Or (95% CI)
Never smoker	300/2527	52.4	73.2	Not applicable	Not applicable	1.00	1.00
Quitting at age <40 years	35/366	51.3	71.9	33.9	37.9	0.94 (0.63–1.35)	0.86 (0.57–1.25)
Quitting at age 40–50 years	50/340	54.4	75.1	44.9	30.2	0.93 (0.66–1.29)	0.84 (0.59–1.18)
Quitting at age 50–60 years	53/336	53.3	74.2	54.9	19.3	1.12 (0.79–1.55)	1.05 (0.74–1.48)
Quitting at age >60 years	79/358	55.3	76.6	66.6	10.0	1.27 (0.95–1.70)	1.20 (0.87–1.63)
Persistent smoker	112/647	52.3	73.2	Not applicable	Not applicable	1.43 (1.11–1.84)	1.34 (1.01–1.76)

Model 1 was adjusted for age at frailty assessment, dialect group, and level of education.

Model 2 was further adjusted for body mass index (baseline, follow-up 3, categorical), alcohol drinking (baseline, follow-up 3, categorical), physical activity (baseline), sleep duration (baseline, follow-up 3, categorical), DASH (baseline, follow-up 3 in ordinal quartiles), caffeine (baseline, in ordinal quartiles), and medical history of hypertension, diabetes, cardiovascular diseases (baseline, follow-up 3).

Sensitivity analyses produced similar overall findings. After reclassifying participants who had quit within 2 years and combining with persistent smokers, the adjusted OR (95% CI) was 1.35 (1.03–1.77) for the combined group and 1.28 (0.81–1.97) for the remaining participants who had quit for <10 years (Supplementary Table S2).

After exclusion of participants with hypertension, diabetes, ischemic heart disease, or stroke at baseline, the corresponding ORs were 1.31 (0.96–1.78) for persistent smokers and 1.49 (0.90–2.38) for those who had quit for <10 years (Supplementary Table S3). With frailty defined as at least 3 of 4 components, the OR for persistent smokers was 1.60 (0.97–2.59), while estimates among former smokers remained imprecise (Supplementary Table S4).

## 4 Discussion

In this prospective population-based cohort study with about 20 years of follow-up, persistent smokers had higher risk of late-life physical frailty than never smokers. While former smokers who had quit for less than 10 years still had a non-significant increased risk estimate close to that of persistent smokers, quitting for at least 10 years before frailty assessment was associated with little to no excess frailty risk. Stopping smoking before age of 60 years brought frailty risk close to that of never smokers, while those who quit after age 60 years still had a non-significant 20 percent increase in risk. Overall, the findings suggest that earlier and sustained smoking cessation may mitigate the elevated risk of late-life physical frailty.

Although previous case-control and cross-sectional studies have documented a link between smoking cessation and physical frailty (Song et al., 2024; Thompson et al., 2018), these designs are susceptible to inherent recall and temporal biases. To date, only a limited number of prospective studies have investigated this relationship between smoking cessation and the risk of late-life frailty.

Findings from 909 community-dwelling older adults in the North West Adelaide Health Study showed that, compared with never smokers, current smokers had substantially higher odds of frailty, whereas the association was attenuated and not evident among former smokers (Thompson et al., 2018). Similarly, the Survey on Health, Ageing, and Retirement in Europe reported worsening frailty among current smokers relative to never smokers, but not among former smokers (Etman et al., 2015). However, neither study further examined whether frailty risk among former smokers differed by age at cessation or by time since quitting before frailty assessment.

The English Longitudinal Study of Ageing investigated the associations of smoking cessation with the subsequent trajectory of frailty status among 2,600 former smokers, and reported that those with a longer duration of quitting smoking had a significantly lower degree of frailty at baseline and over time. Interestingly, although those who quit 41 years earlier or more had the lowest frailty trajectory, there was still a gap between them and never smokers (Kojima et al., 2025). However, smoking status was measured only once at baseline, and former smokers were classified according to their self-report at that time. As such, participants who quit before baseline but later relapsed could have been misclassified as former smokers during the study, which may have inflated frailty risk estimates among former smokers compared to never smokers.

There are several postulated mechanisms underlying the relationship between cigarette smoking and physical frailty. Numerous studies have demonstrated a higher level of inflammatory markers in chronic smokers, including C-reactive protein and the pro-inflammatory cytokines interleukin-1β and interleukin-6 (Ugur et al., 2018; Toker et al., 2012). Recently, the term inflamm-ageing (Pan and Ma, 2024; Fulop et al., 2017; Franceschi et al., 2017) has been coined to describe the link between chronic inflammation and ageing-related biomechanistic processes, including cellular senescence, systemic organ dysfunction, reduced physiological reserves (Pan and Ma, 2024; Fulop et al., 2017; Ferrucci and Fabbri, 2018; Leonardi et al., 2018; Salimi et al., 2018; Gorelick, 2010; Lee et al., 2026), and comorbidities such as cardiovascular (Ambrose and Barua, 2004) and respiratory diseases (Aghapour et al., 2018), all of which have been implicated in the development of frailty. These inflammatory markers have been shown to decline after smoking exposure is eliminated (Peres et al., 2017; Elisia et al., 2020), which may explain our finding that individuals who had quit smoking for a sufficiently long period had frailty risk comparable to that of never smokers. Finally, since early quitting automatically reduces total lifetime smoking, the health benefits observed after early cessation could simply be explained by reduced cumulative exposure. As such, one of the reasons quitting early is beneficial could be that it reduces the total amount of smoking a person accumulates over life.

The strengths of our study are its long follow-up duration, comprehensive adjustment of potential confounding covariates, and meticulous and repeated ascertainment of smoking status at baseline and over three follow-up visits spanning average 20 years, which allowed us to accurately classify participants as persistent, former and never smokers, and exclude those who relapsed after quitting.

However, this study had several limitations. First, the modified frailty phenotype excluded low physical activity and classified participants meeting at least 2 of 4 available criteria as frail. This could have classified some participants who would have been considered pre-frail using the original five-component phenotype as frail. In the sensitivity analysis using a stricter definition of at least 3 of 4 criteria, the estimate for persistent smoking remained elevated, although the risk estimate did not reach statistical significance due to reduced number in frailty outcome. Second, we acknowledge that our study only included a portion of the original populations who survived and consented to follow-up 3 interviews, which would have introduced a selection bias of only including the healthier participants from the initial study sample. As shown in Supplementary Table S5, compared with the 23,380 men who were not included in this study, the 4,574 men in this study were younger, had higher educational attainment, greater physical activity, and lower baseline prevalence of hypertension, diabetes, and heart attack at enrolment (1993–1998) than those who were not included. Such inclusion of participants with a lower baseline frailty risk could have biased the exposure-outcome association toward the null and led to an underestimation of the risk estimates. Third, since we only conducted frailty assessment at the third follow-up, reverse causality could not be precluded since former smokers could have developed frailty before smoking cessation. Alternatively, long-term abstainers may represent a selectively healthier or more health-conscious group. Finally, a previous Chinese cohort reported a stronger association between smoking and frailty in men than in women (Wang et al., 2013), and since we only studied men, our findings may not be generalized to women.

## 5 Conclusion

In conclusion, our study adds prospective evidence that smoking is an important and potentially modifiable risk factor for physical frailty in later life, and that quitting smoking for at least 10 years (or before age 60 years) may substantially mitigate this excess frailty risk to levels close to never smokers. The results are biologically plausible in light of smoking-related systemic inflammation and its downstream effects on age-related physiological vulnerability, and they underscore the potential benefits of quitting earlier and sustaining cessation. Future studies with earlier and repeated frailty assessments, careful handling of time-varying smoking behaviors, and more diverse populations are needed to further clarify temporality, reduce residual bias, and strengthen the generalizability of these observations.

## Data Availability

The raw data supporting the conclusions of this article will be made available by the authors, without undue reservation.
