# Supplementary material for: Association of smoking cessation with late-life frailty risk: the Singapore Chinese health study
Source: Front Aging. 2026 Sep 17;7:1870256. doi: 10.3389/fragi.2026.1870256 (PMC13627434; doi:10.3389/fragi.2026.1870256)
Supplement: Supplementary file 1 [file Table1.docx]

**Supplementary Table 1. Test for trend across smoking-cessation duration among former smokers.**

| Trend analysis | N | Cases | OR per category | 95% CI | P value |
| --- | --- | --- | --- | --- | --- |
| Ordered cessation-duration trend among former smokers using Model 1 | 1400 | 217 | 0.90 | 0.80-1.02 | 0.106 |
| Ordered cessation-duration trend among former smokers using Model 2 | 1400 | 217 | 0.90 | 0.78-1.03 | 0.112 |

Ordinal scores were assigned to categories of increasing duration since cessation (<10, 10-20, 20-30, 30-40, and >40 years).

**Supplementary Table 2. Associations with late-life physical frailty after reclassification of participants who quit within 2 years as persistent smokers.**

| Panel | Smoking category | Cases/total | Model 1 OR (95% CI) | Model 2 OR (95% CI) |
| --- | --- | --- | --- | --- |
| Duration since smoking cessation | Never smoker | 300/2527 | 1.00 | 1.00 |
|  | Quitting >40 years | 26/146 | 1.02 (0.63-1.60) | 0.97 (0.59-1.55) |
|  | Quitting 30-40 years | 51/385 | 0.93 (0.66-1.29) | 0.85 (0.60-1.19) |
|  | Quitting 20-30 years | 49/328 | 1.01 (0.71-1.42) | 0.91 (0.63-1.29) |
|  | Quitting 10-20 years | 55/340 | 1.12 (0.80-1.55) | 1.08 (0.76-1.51) |
|  | Quitting <10 years | 31/177 | 1.41 (0.90-2.13) | 1.28 (0.81-1.97) |
|  | Persistent smoker or quit ≤2 years | 117/671 | 1.45 (1.13-1.86) | 1.35 (1.03-1.77) |
| Age at smoking cessation | Never smoker | 300/2527 | 1.00 | 1.00 |
|  | Quitting at age <40 years | 35/366 | 0.94 (0.63-1.35) | 0.86 (0.57-1.25) |
|  | Quitting at age 40-50 years | 50/340 | 0.93 (0.66-1.29) | 0.84 (0.59-1.18) |
|  | Quitting at age 50-60 years | 53/336 | 1.12 (0.79-1.55) | 1.05 (0.74-1.48) |
|  | Quitting at age ≥60 years | 74/334 | 1.24 (0.91-1.67) | 1.17 (0.84-1.60) |
|  | Persistent smoker or quit ≤2 years | 117/671 | 1.45 (1.12-1.85) | 1.35 (1.03-1.77) |

Model 1 was adjusted for age at frailty assessment, dialect group, and level of education.

Model 2 was further adjusted for body mass index (baseline, follow-up 3, categorical), alcohol drinking (baseline, follow-up 3, categorical), physical activity (baseline), sleep duration (baseline, follow-up 3, categorical), DASH (baseline, follow-up 3 in ordinal quartiles), caffeine (baseline, in ordinal quartiles), and medical history of hypertension, diabetes, cardiovascular diseases (baseline, follow-up 3).

**Supplementary Table 3. Associations with late-life physical frailty after exclusion of participants with baseline hypertension, diabetes, ischemic heart disease, or stroke.**

| Panel | Smoking category | Cases/total | Model 1 OR (95% CI) | Model 2 OR (95% CI) |
| --- | --- | --- | --- | --- |
| Duration since smoking cessation | Never smoker | 187/1921 | 1.00 | 1.00 |
|  | Quitting >40 years | 14/93 | 1.13 (0.59-2.02) | 1.05 (0.54-1.92) |
|  | Quitting 30-40 years | 30/260 | 0.97 (0.62-1.46) | 0.92 (0.59-1.41) |
|  | Quitting 20-30 years | 22/224 | 0.77 (0.46-1.22) | 0.76 (0.45-1.22) |
|  | Quitting 10-20 years | 35/263 | 1.04 (0.69-1.55) | 1.02 (0.66-1.54) |
|  | Quitting <10 years | 27/161 | 1.67 (1.04-2.62) | 1.49 (0.90-2.38) |
|  | Persistent smoker | 89/570 | 1.52 (1.13-2.02) | 1.31 (0.96-1.78) |
| Age at smoking cessation | Never smoker | 187/1921 | 1.00 | 1.00 |
|  | Quitting at age <40 years | 23/252 | 1.09 (0.67-1.71) | 0.99 (0.60-1.58) |
|  | Quitting at age 40-50 years | 21/224 | 0.71 (0.43-1.14) | 0.70 (0.41-1.12) |
|  | Quitting at age 50-60 years | 29/250 | 0.97 (0.62-1.48) | 0.95 (0.60-1.47) |
|  | Quitting at age ≥60 years | 55/275 | 1.37 (0.96-1.94) | 1.31 (0.90-1.88) |
|  | Persistent smoker | 89/570 | 1.52 (1.13-2.02) | 1.31 (0.96-1.79) |

Model 1 was adjusted for age at frailty assessment, dialect group, and level of education.

Model 2 was further adjusted for body mass index (baseline, follow-up 3, categorical), alcohol drinking (baseline, follow-up 3, categorical), physical activity (baseline), sleep duration (baseline, follow-up 3, categorical), DASH (baseline, follow-up 3 in ordinal quartiles), caffeine (baseline, in ordinal quartiles), and medical history of hypertension, diabetes, cardiovascular diseases (baseline, follow-up 3).

**Supplementary Table 4. Associations using frailty defined as at least 3 of 4 available components.**

| Panel | Smoking category | Cases/total | Model 1 OR (95% CI) | Model 2 OR (95% CI) |
| --- | --- | --- | --- | --- |
| Duration since smoking cessation | Never smoker | 70/2527 | 1.00 | 1.00 |
|  | Quitting >40 years | 10/146 | 1.67 (0.78-3.23) | 1.60 (0.72-3.20) |
|  | Quitting 30-40 years | 12/385 | 0.94 (0.47-1.70) | 0.92 (0.46-1.68) |
|  | Quitting 20-30 years | 15/328 | 1.37 (0.74-2.39) | 1.28 (0.67-2.31) |
|  | Quitting 10-20 years | 14/340 | 1.23 (0.65-2.17) | 1.24 (0.64-2.25) |
|  | Quitting <10 years | 8/201 | 1.36 (0.59-2.76) | 1.15 (0.48-2.41) |
|  | Persistent smoker | 31/647 | 1.72 (1.08-2.67) | 1.60 (0.97-2.59) |
| Age at smoking cessation | Never smoker | 70/2527 | 1.00 | 1.00 |
|  | Quitting at age <40 years | 8/366 | 1.00 (0.44-2.00) | 0.95 (0.41-1.92) |
|  | Quitting at age 40-50 years | 11/340 | 0.86 (0.42-1.60) | 0.78 (0.38-1.48) |
|  | Quitting at age 50-60 years | 18/336 | 1.68 (0.94-2.84) | 1.74 (0.96-3.03) |
|  | Quitting at age ≥60 years | 22/358 | 1.44 (0.85-2.36) | 1.36 (0.78-2.30) |
|  | Persistent smoker | 31/647 | 1.72 (1.09-2.68) | 1.63 (0.99-2.64) |

Model 1 was adjusted for age at frailty assessment, dialect group, and level of education.

Model 2 was further adjusted for body mass index (baseline, follow-up 3, categorical), alcohol drinking (baseline, follow-up 3, categorical), physical activity (baseline), sleep duration (baseline, follow-up 3, categorical), DASH (baseline, follow-up 3 in ordinal quartiles), caffeine (baseline, in ordinal quartiles), and medical history of hypertension, diabetes, cardiovascular diseases (baseline, follow-up 3).

**Supplementary Table 5. Comparison of baseline characteristics (1993-1998) between cohort participants who were included and not included in the study**

| **Characteristic** | **Included in the study (n=4,574)** | **Not included in the study (n=23,380)** | **P value** |
| --- | --- | --- | --- |
| Number of participants | 4574 | 23380 | - |
| Age at baseline, y | 52.7 ± 5.1 | 57.6 ± 8.1 | <0.001 |
| BMI, kg/m² | 23.2 ± 3.2 | 22.9 ± 3.2 | <0.001 |
| Dialect group |  |  | <0.001 |
| Hokkien | 2325 (50.8) | 13289 (56.8) |  |
| Cantonese | 2249 (49.2) | 10091 (43.2) |  |
| Level of education |  |  | <0.001 |
| No formal education | 169 (3.7) | 2893 (12.4) |  |
| Primary | 1979 (43.3) | 12329 (52.7) |  |
| Secondary or higher | 2426 (53.0) | 8158 (34.9) |  |
| Caffeine intake, mg/day | 158.0 ± 112.1 | 163.3 ± 114.0 | 0.004 |
| Alcohol consumption |  |  | <0.001 |
| Non-drinker | 3009 (65.8) | 16243 (69.5) |  |
| Monthly drinker | 601 (13.1) | 2348 (10.0) |  |
| Weekly drinker | 736 (16.1) | 3218 (13.8) |  |
| Daily drinker | 228 (5.0) | 1571 (6.7) |  |
| Sleep duration |  |  | <0.001 |
| ≤5 hours | 261 (5.7) | 2129 (9.1) |  |
| 6-8 hours | 4111 (89.9) | 19484 (83.3) |  |
| ≥9 hours | 202 (4.4) | 1767 (7.6) |  |
| Physical activity |  |  | <0.001 |
| No physical activity | 2243 (49.0) | 13648 (58.4) |  |
| 0.5-3.9 hours a week | 1437 (31.4) | 5685 (24.3) |  |
| ≥4 hours/week | 894 (19.5) | 4047 (17.3) |  |
| Hypertension |  |  | <0.001 |
| No | 3695 (80.8) | 17704 (75.7) |  |
| Yes | 879 (19.2) | 5676 (24.3) |  |
| Diabetes |  |  | <0.001 |
| No | 4351 (95.1) | 21178 (90.6) |  |
| Yes | 223 (4.9) | 2202 (9.4) |  |
| History of heart attack |  |  | <0.001 |
| No | 4468 (97.7) | 22117 (94.6) |  |
| Yes | 106 (2.3) | 1263 (5.4) |  |

Baseline comparison data were available for 27,954 of the 27,959 men recruited at baseline. Data are mean ± SD or n (%). Between-group differences were assessed using one-way ANOVA for continuous variables and the chi-square test for categorical variables.
